# Compliance with Nutritional Recommendations and Gut Microbiota Profile in Galician Overweight/Obese and Normal-Weight Individuals

**DOI:** 10.3390/nu15153418

**Published:** 2023-08-01

**Authors:** Laura Sinisterra-Loaiza, Patricia Alonso-Lovera, Alejandra Cardelle-Cobas, Jose Manuel Miranda, Beatriz I. Vázquez, Alberto Cepeda

**Affiliations:** Laboratorio de Higiene, Inspección y Control de Alimentos, Departamento de Química Analítica, Nutrición y Bromatología, Campus Terra, Universidade da Santiago de Compostela, 27002 Lugo, Spain; laura.sinisterra@usc.es (L.S.-L.); patricia.alonso.lovera@rai.usc.es (P.A.-L.); josemanuel.miranda@usc.es (J.M.M.); beatriz.vazquez@usc.es (B.I.V.); alberto.cepeda@usc.es (A.C.)

**Keywords:** gut microbiota, obesity, fiber, monounsaturated fatty acids, BMI

## Abstract

Different research studies have identified specific groups or certain dietary compounds as the onset and progression of obesity and suggested that gut microbiota is a mediator between these compounds and the inflammation associated with pathology. In this study, the objective was to evaluate the dietary intake of 108 overweight (OW), obese (OB), and normal-weight (NW) individuals and to analyze their gut microbiota profile to determine changes and associations with Body Mass Index (BMI) and diet. When individuals were compared by BMI, significant differences in fiber and monounsaturated fatty acids (MUFAs) intake were observed, showing higher adequacy for the NW group. The analysis of gut microbiota showed statistical differences for 18 ASVs; *Anaerostipes* and *Faecalibacterium* decreased in the OW/OB group, whereas the genus *Oscillospira* increased; the genus was also found in the LEFSe analysis as a biomarker for OW/OB. *Roseburia faecis* was found in a significantly higher proportion of NW individuals and identified as a biomarker for the NW group. Correlation analysis showed that adequation to nutritional recommendation for fiber indicated a higher abundance of *Prevotella copri*, linearly correlated with *F. prausnitzii*, *Bacteroides caccae*, and *R. faecis*. The same correlation was found for the adequation for MUFAs, with these bacteria being more abundant when the intake was adjusted to or below the recommendations.

## 1. Introduction

Western societies have undergone a process that involves major qualitative and quantitative changes in dietary habits. Thus, traditional diets have been replaced by diets characterized by a higher energy load and a decrease in fiber and complex carbohydrates. These changes, in addition to behavioral changes such as less physical activity, have resulted in an increase in worldwide overweight and obesity rates [1]. Obesity is the abnormal or excessive accumulation of fat that can be detrimental to health. It is of multifactorial origin, resulting from pathological processes and deriving from an interrelation between numerous factors [2,3].

According to the European Health Survey in Spain 2020, about 16% of the Spanish population suffers from obesity, while 37.60% of the population is overweight [4]. Other recent monitoring work revealed that Galicia is one of the regions of Spain in which there are higher rates of obesity in adults, reaching 26.7% [5]. Additionally, a study reported that during the COVID-19 confinement, 44% of participants indicated an increase in their body weight, with an average increase of 2.8 kg [6]. Thus, it is probably true that the current overweight rate of Galician people could now be higher than those in the cited reports [4,5].

Several authors have pointed out that the dietary habits of Galician people do not follow the same patterns as in the rest of Spain. However, together with the north of Portugal, they are included in the dietary model known as the Atlantic Diet [7,8]. Knowledge of the dietary patterns of the population is essential to understanding the potential impact of the strategies implemented to prevent the increase in obesity rates [9].

One approach to treating obesity in recent years involves the study of the gut microbiota, the results of which in both mice and humans describe remarkable differences between the gut microbiota of obese (OB) and normal-weight (NW) subjects. When there is an excess of body fat, colonic concentrations of Firmicutes increase by more than 50%, while those of Bacteroides decrease correlatively compared to NW subjects [10]. The composition of the gut microbiota of obese individuals is characterized by a decrease in genera such as *Akkermansia*, *Alistipes*, *Faecalibacterium*, and *Oscillibacter* [11,12] and an increase in *Staphylococcus* and *Clostridium* [13,14,15,16].

In addition, dietary patterns are considered modulators of the gut microbiota; for example, excessive fat consumption induces an imbalance in the gut microbiota, leading to gut barrier dysfunction, increased host body weight, and low-grade inflammation of adipose tissue [17]. A high-protein diet, on the other hand, increases the growth of bile-tolerant bacterial species (*Alistipes*, *Bilophila*, and *Bacteroides*) and decreases bacteria that hydrolyze disaccharides or polysaccharides into simple carbohydrates (*Roseburia*, *Eubacterium rectale*, and *Ruminococcus bromii*) [18].

Although there is available evidence about the differences in gut microbiota composition in both overweight or obese and normal-weight individuals, there is scarce information about potential biomarkers associated with certain dietary compounds. Thus, the hypothesis of the present study is that dietary intake, especially specific dietary components, is associated with different gut microbiota compositions in both overweight or obese and normal-weight subjects. Therefore, the aim of this study was to differentiate the gut microbiota of overweight (OW) and OB subjects from that of NW subjects, in a sample of Galician people, compare the results with previous studies to establish potential biomarkers and determine its association with dietary components potentially diminished or increased in each group under study.

## 2. Materials and Methods

### 2.1. Population and Sample Size

This cross-sectional study is part of an international project about the search for novel biomarkers in diabetes and obesity in Iberian-America (CyTED project 918PTE0540). The study population for the present analysis included 108 individuals, ranging in age from 40 to 70 years, with habitual residence in Galicia (a northwest region of Spain). The sample size was estimated based on previous studies [19,20,21]. The participants were informed of the objectives of the study, and informed consent was obtained from each volunteer to participate in the study, and all data obtained were handled according to Spanish Law 3/2018 on personal data protection. This consent detailed the conditions of the research, highlighting the voluntary nature of participation, the possibility of withdrawing from the study even with the acceptance of the consent, and the anonymous treatment of the data for research purposes only.

The following inclusion criteria were used: age between 40 and 70 years; absence of diagnosed pathologies, not having undergone medical treatment with hormones, corticoids, or having recently consumed any of the following substances: proton pump inhibitors, amphetamines, alpha-adrenergic drugs, alpha-blockers, beta-blockers, opiates, calcium-antagonists, neuroleptics, tricyclic antidepressants, phenothiazines, central nervous system stimulants (cocaine, etc.); not having consumed any supplement containing probiotics or prebiotics during the previous 2 months; in the case of women, not being pregnant

Ethical approval for this study was obtained from The Regional Ethics Committee for Clinical Research (Galician Health Service, SERGAS, n° 2018/270) in compliance with the Declaration of Helsinki of 1964 regarding privacy, confidentiality, and informed consent. All experiments were carried out in accordance with approved guidelines and regulations.

### 2.2. Anthropometric Measures

Anthropometric measurements were obtained. Weight was determined using an InBody 127 digital scale (InBody, Tokyo, Japan). Height was measured with a portable stadiometer (ADE MZ10042; Hamburg, Germany), with the subject upright and in balance, without bending the knees. Subsequently, the Body Mass Index (BMI) was calculated using the Quetelet formula: BMI = weight (kg)/[height (m)]^2^. According to the classification ranges proposed by the Spanish Obesity Society [22], three volunteers were grouped in groups according to their BMI: 18–24.9 kg/m^2^ for NW, 25–29.9 kg/m^2^ for OW, and ≥30 OB kg/m^2^.

### 2.3. Dietary Information

A 72 h dietary record was completed by the volunteers for 3 days (2 workdays and 1 weekend day). The volunteers were given instructions on how to record their dietary intake. They were also asked to provide information on the portions of food consumed, the ingredients and techniques used in cooking, and the type and quantity of beverages consumed.

The mean daily energy and nutrient intake of each volunteer were calculated using the open software “diet calculator”, available on the website of the Endocrinology and Clinical Nutrition Research Centre [23], which is based on Spanish foods. Data obtained from the software were compared to the nutritional objectives for the Spanish adult population published by the Spanish Society of Community Nutrition (SENC, 24) to determine their nutritional adequacy. The data obtained were compared by sex and by BMI of volunteers.

Data about micronutrient (minerals and vitamins) intake were compared to Reference Dietary Intakes (RDI) established by the Spanish Federation of Nutrition, Food and Dietetics Societies [24]. The data obtained were compared by sex and BMI of the volunteers.

### 2.4. Fecal Sample Collection and DNA Extraction

Volunteers received detailed instructions to collect fecal samples and were provided with a sterile container that should deliver to the laboratory along with the 72 h dietary record. Samples should have been delivered within two hours after defecation or, in the case of not being able to do it, they should have been immediately frozen at −20 °C after deposition and delivery to the laboratory where they were conserved frozen until treated for analysis.

DNA from fecal samples was extracted using the Dneasy Powersoil kit (Qiagen^®^, Hilden, Germany) following the manufacturer’s instructions. Extracted DNA was then quantified using a Qubit™4 fluorometer (Invitrogen, Thermo Fisher Scientific, Carlsbad, CA, USA) and the Qubit kit 1X dsDNA High Sensitivity (Thermo Fisher Scientific, Inc Alemania). After quantification, DNA samples were frozen and stored at −20 °C until further analysis.

### 2.5. 16S rRNA Amplicon Sequencing

For 16S rRNA amplicon sequencing, 2 μL of DNA extracted from each sample was used to construct the libraries, and the Ion GeneStudio^TM^ S5 System (Life Technologies, Carlsbad, CA, USA) was used. For this purpose, the 16S hypervariable regions were amplified with two sets of primers, v2–4–8 and v3–6, 7–9, and the libraries were prepared by using the Ion 16S^TM^ Metagenomics Kit (Life Technologies) and the Ion Xpress^TM^ Plus Fragment Library Kit (Life Technologies). Libraries containing equal amounts of PCR products pooled with a barcode were prepared by using the Ion Xpress^TM^ Barcode Adapters Kit (Life Technologies). Then, these libraries were quantified by using the Ion Universal Library Quantitation Kit (Life Technologies). Next, 10 pM of each library was pooled and loaded on an Ion OneTouch™ 2 System (Life Technologies), which automatically performs template preparation and enrichment. Template-positive ion sphere particles were enriched with Dynabeads™ MyOne™ Streptavidin C1 magnetic beads (Invitrogen, Carlsbad, CA, USA) by using an Ion One Touch ES instrument. Finally, an Ion 520 TM chip (Life Technologies) was loaded with the samples on an Ion GeneStudioTM S5 System sequencer using the Ion 520™ & Ion 530™ Loading Reagents supplied in the OT2-Kit (Life Technologies).

### 2.6. Statistical and Bioinformatic Analysis

Student’s t-test for independent samples was used to compare qualitative variables between different groups (sex or BMI). The X2 test with Yates’s correction was used to compare frequencies. In all cases, the obtained differences were considered statistically significant if the *p* value was less than 0.05.

A two-way ANOVA was used to determine significative differences for time and substrates as the two covariates in the general linear model using Tukey’s analysis. For significant differences (*p* < 0.05) a one-way ANOVA was conducted for each substrate comparing 0, 5, 10, and 24 h. Similarly, each substrate was compared using two-way ANOVA; results were significant when *p* < 0.05. The software SPSS^®^ v.27 for Windows (SPSS Inc., Chicago, IL, USA) was used for these analyses.

For the analysis of 16S rRNA amplicon sequencing, the raw sequencing reads were obtained from the Torrent Suite software (v. 5.12.2.) as fastq files. The fastq files were processed with QIIME 2 software v. 2022.11 [25]. To produce amplicon sequence variants (ASVs), the DADA2 method was used for quality filtration (Q score ≥ 30), trimming, denoising, and dereplication. Samples with features (taxa) with a total abundance (summed across all samples) of <10 were removed. Taxonomy was assigned to ASVs by using the q2-feature-classifier classify-sklearn naïve Bayes taxonomy classifier, which was compared to the Greengenes 13_8 99% operational taxonomic unit (OTU) reference sequences.

STAMP software (v 2.1.3) for the “Statistical Analysis of Taxonomic and Functional Profiles” [26] was used to determine statistical differences in the obtained ASVs at the species level. Kruskal–Wallis H test with post hoc Tukey–Kramer test was employed.

The OTUs with taxonomic information, obtained in QIIME2, were used together with a metadata file, in text format, for their analyses in the free platform Microbiome Analyst, “a comprehensive statistical, functional and integrative analysis of microbiome data” [27]. alpha- and beta- diversity, correlation, and LEfSe analysis were carried out.

## 3. Results

### 3.1. Nutritional Analysis and Adequation to the Objectives and the Recommended Daily Intakes for the Spanish Population

A total of 108 subjects completed a 72 h dietary recall, and their anthropometric measurements were obtained. Of these, 67 (62%) were women and 41 (38%) were men. The average age of the subjects was 51.0 ± 8.0 years, and the average kilocalorie intake was 2344.0 ± 556.9 kcal/day. Comparing this energy intake by sex, a significantly higher intake (*p* = 0.04) was observed for men (2502.6 ± 538.3) than for women (2247.0 ± 537.0). Data obtained for macronutrients and other parameters are shown in Table 1.

As it can be observed, the average total carbohydrate intake in terms of % of energy is deficient, 88.1% of the individuals do not achieve the recommendations. The same occurs for fiber intake (84.3% of individuals are in deficit). Regarding the lipid profile, 89.6% of the participants are above the objectives, which indicates a diet rich in fat, especially in saturated fat since 88% of the volunteers showed to possess a consumption above the recommendations. For protein, it is necessary to indicate that this macronutrient does not appear in the recommendations for the Spanish population, since its determination is depending on the other two macronutrients. The percent for protein usually recommended is 10–15% of total energy. The data obtained for the participants of this study indicated an adequation of 38.9% and an excess of 61.1%.

For simple sugar, only 13 individuals showed to have a consumption below a 10%, being an average intake of 18.1 ± 7.4%. Regarding fruit and vegetable consumption, the average intake of fruit and vegetables was 1.8 ± 1.5 servings, which is <50% of the recommended five daily servings; only 4.6% of the population surveyed had the recommended intake, while 94.4% did not meet the recommendations. Regarding other parameters such as alcohol consumption, most of the volunteers showed to be below the recommendation of 1 and 2 Standard Drink Units (SDUs) of alcoholic beverages, for women and men, respectively. Regarding water consumption, 45.4% of the participants did not reach the recommendations for water consumption.

When the data obtained were compared by sex (Table S1), there were some significant differences in dietary patterns between men and women.

Thus, in general, women accomplish more nutritional objectives than men. On average, men have a higher intake of protein, a lower consumption of carbohydrates, and a higher consumption of fat than women. Regarding the lipid profile, statistical differences were found for SFA, where women, once again, showed to better accomplish the nutritional objectives for the Spanish population.

Comparing intake data according to BMI (Table 2), no statistical differences were found among NW, OW, and OB subjects for all parameters investigated with the exception of total energy, fiber, and MUFAs, for which statistical differences were found. In the case of fiber, a better adequation (32.5%) was observed for NW individuals than for OW/OB (15.2 and 14.3, respectively). For MUFAs, only 14.3% of the OB volunteers accomplish the objectives, whereas for the OW and NW groups, the accomplishment was higher, 36.4 and 25%, respectively. For total energy, 2284.0 ± 658.6 kcal/day were obtained for the NW group, whereas for the OW and OB group, the total energy obtained was 2221.3 ± 497.2 and 2498.4 ± 459.1 kcal/day, respectively. A significantly higher caloric intake was obtained for the OB group (*p* = 0.0356).

Regarding water, fruit, vegetables, and sugar, no statistical differences were observed according to BMI. However, in general, the NW and the OW groups showed higher adequacy for water and alcohol consumption. For sugar consumption, the worst adequacy was obtained for the NW group, but these values included all the simple sugar consumed, including that provided for fruits and not only the added simple sugar, and this is the group consuming more fruits and vegetables.

Regarding the intake of micronutrients, a comparison between women and men can be seen in Table S2. Significant differences were found between sexes for the intake of thiamine, riboflavin, niacin, vitamin B6, vitamin E, phosphorus, and iron, with the average daily intake being higher in men than in women for all the micronutrients. Regarding adequacy, however, women possess a major percentage of adequacy than men.

When comparing micronutrient intake by BMI (Table 3), it was found that NW subjects consumed higher amounts of some micronutrients such as iron, iodine, and ascorbic acid than OW and OB patients, whereas tocopherol intake was significantly higher for OW subjects and OB patients, and a higher intake of calciferol with respect to both NW and OW subjects was observed. Compliance with intake with current recommendations was higher in NW subjects than in OW subjects for calcium, iron, iodine, zinc, ascorbic acid, and calciferol. Only in the case of magnesium were the highest compliance rates found in OB subjects with respect to NW and OW patients.

### 3.2. Analysis of the Gut Microbiota Composition

#### 3.2.1. Alpha- and Beta-Diversity

A total of 95 fecal samples were collected from the volunteers. A first analysis between groups (NW, OW, and OB) was carried out; however, no differences were obtained between the OW and OB groups. Therefore, a second analysis conducted by grouping OW and OB was developed. Although the BMI is the most extended method, in clinical practice, to classify overweight and obesity in adults, it is not the most adequate to determine the amount of body fat. In addition, the nutritional analysis has shown that, in terms of adequacy, the individuals included in the NW group accomplish in higher proportion the nutritional objectives and the DRI for the Spanish population than the OW and OB groups (for example, the % of adequacy for the group of OW and OB is 0, whereas 15% of the individuals in the NW group meet the RDI)

**Figure 1:**
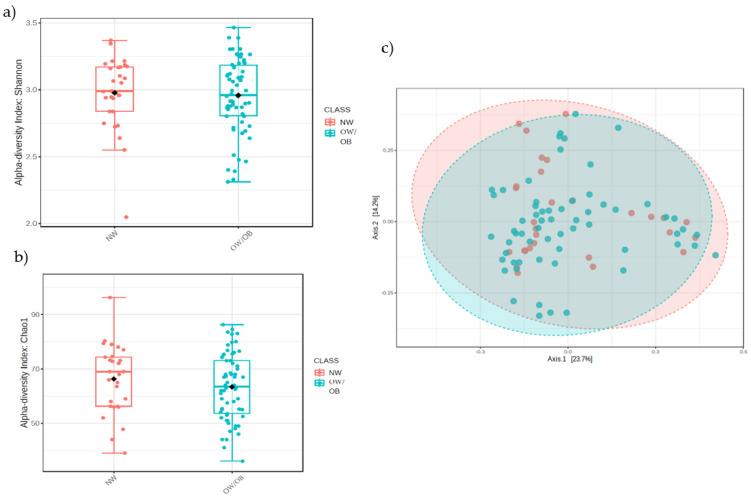
(**a**) Shannon index and (**b**) Chao1 index (alpha-diversity), in red for normal-weight individuals (NW) and, in blue for overweight/obese (OW/OB); (**c**) beta-diversity determined by principal coordinates analysis (PCoA) based on the Bray-Curtis dissimilarity index; red NW group and blue OW/OB group.

To investigate alpha-diversity, the Chao1 richness and Shannon diversity (richness and abundance) indices were determined. No statistical differences were found between the NW and OW/OB groups for Chao1 (ANOVA, *p* = 0.1953) nor for Shannon (*p* = 0.711). For beta-diversity, calculated by Bray-Curtis, no statistical differences were found between groups (F-value: 0.7111; R-squared: 0.0079266; *p*-value: 0.768) either. See Figure 1.

#### 3.2.2. Relative Abundance of Bacteria

**Figure 2:**
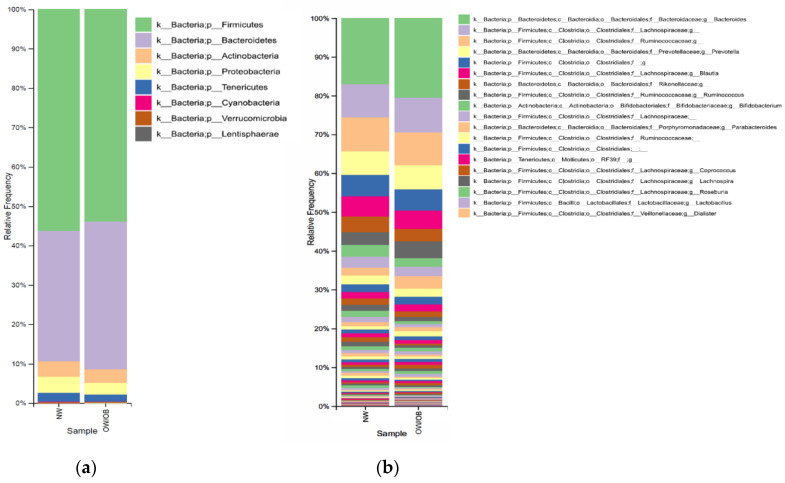
**Relative abundance of different bacterial phyla and genera**. Bacterial composition (relative abundance, %) was determined using 16S rRNA amplicon sequencing at the phylum (**a**) and genus (**b**) levels. The x-axis shows the different groups evaluated (NW vs. OW/OB). Due to the large number of reported bacteria, only the top 19 most abundant genera were included in the legend. NW: normal weight; OW/OB, overweight/obese individuals.

The relative frequency (Figure 2) at the phylum level (a) showed an increase in the Bacteroidetes phlylum in the OW/OB (35.5%) group in comparison with the NW group (33.2%), whereas the Firmicutes phylum decreased in the OW/OB group (53.3% vs. 56.3%) as well as Actinobacteria (3.54 vs. 3.90%). At the genus level (Figure 1b), the main identified bacteria were *Bacteroides* and *Prevotella*, contributing to the Bacteroidetes phylum, while *Blautia* and *Ruminoccocus* formed the Firmicutes phylum, and *Bifidobacterium* contributes to Actinobacteria phylum; in the Proteobacteria phylum, the main genus was *Sutterella* and *Succinivibrio*. Finally, at the species level, 50 species were identified, with the main identified ones being *Prevotella copri*, *Bacteroides uniformis*, *Bifidobacterium adolescentis*, and *Bifidobacterium longum*, among others.

**Figure 3:**
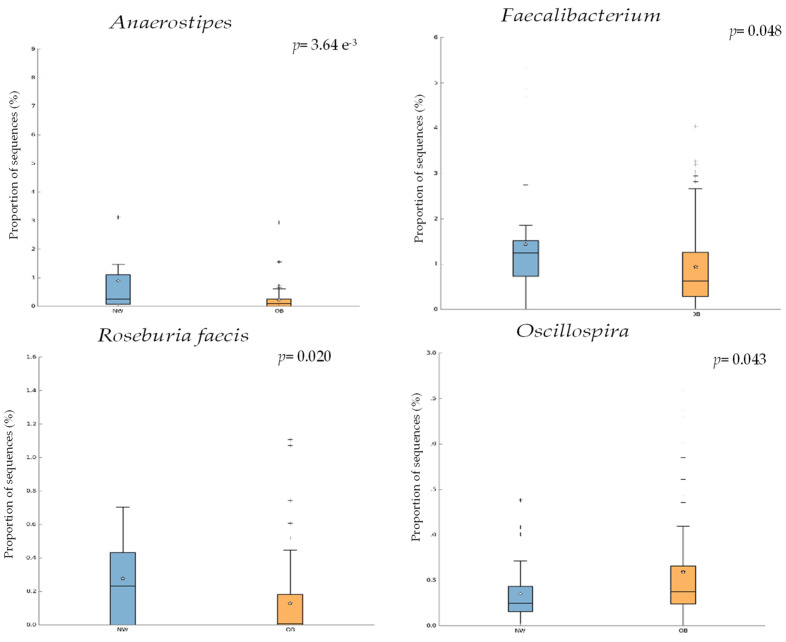
Box plots obtained in the statistical analysis with STAMP for the genera Anaerostipes, *Faecalibacterium*, *Oscillospira*, and the species *R. faecis*. Blue represents the NW group, and orange is the OW/OB group.

Regarding the statistical differences, the analysis using the software STAMP showed statistical differences for 18 ASVs (see table in Supplementary Material). In Figure 3, it is possible to see the box plots for three identified bacterial genera and 1 other species. In the graphics, it can be observed as the genera *Anaerostipes* and *Faecalibacterium* decreased in the OW/OB group whereas the genus Oscillospira increased. For the species, *R. faecis*, the proportion of sequence obtained was statistically higher in NW individuals than in OW/OB.

#### 3.2.3. Correlation Analysis: Microbiota-Fiber, Microbiota-MUFAs

**Figure 4:**
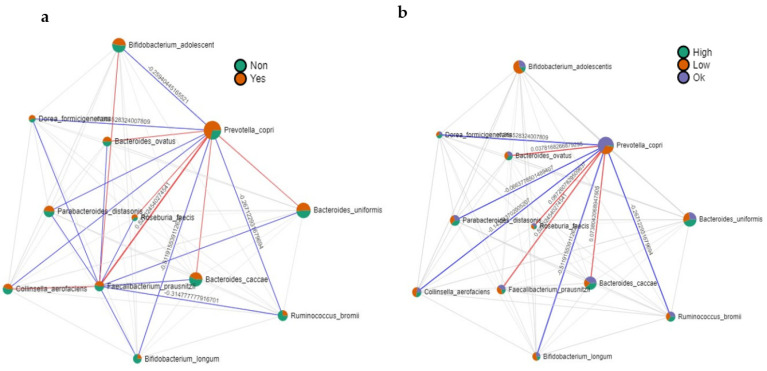
Correlation analysis. Figures show the obtained results for the correlation analysis between identified bacterial species and (**a**) adequation to fiber intake in green, no adequation in red (**b**) MUFAS consumption: above the recommendations(green), below the recommendation(red), and an adequate consumption (purple). Red lines showed a positive correlation among bacterial species, whereas blue lines showed a negative correlation.

Since statistical differences were found for fiber and MUFAs intake in the nutritional analysis, a correlation analysis was carried out with these two factors to establish a linear relationship among the identified species. Individuals were, in this case, classified depending on their adequation to the recommended intake, reaching or not reaching the recommendation standards for an adequate, lower, or high consumption in the case of MUFAs. Figure 4a,b show the obtained results. As can be seen in Figure 4a, *P. copri* was more abundant in individuals achieving recommendations for fiber, and this species was positively correlated with *F. prausnizii*, *B. ovatus*, *B. caccae*, *B. uniformis*, and *R. faecis*. A negative correlation was found for *B. adolescentis*, *Collinsella aerofaciens*, *Dorea formicigenerans*, *Parabacteroides distasonis*, *Ruminococcus bromii*, and *B. longum*. The bacteria positively correlated with fiber were those more abundant in NW individuals (see Supplementary Material Figure S1).

Regarding MUFAs (Figure 4b), *P. copri* was more abundant in those individuals with adequate consumption to recommendations, followed by individuals with low consumption, and the lowest abundance was observed for those individuals with high consumption. For this species, a positive correlation was observed with *B. caccae*, *F. prausnitzii*, *R. faecis*, and *B. ovatus*.

#### 3.2.4. LEfSe Analysis

**Figure 5:**
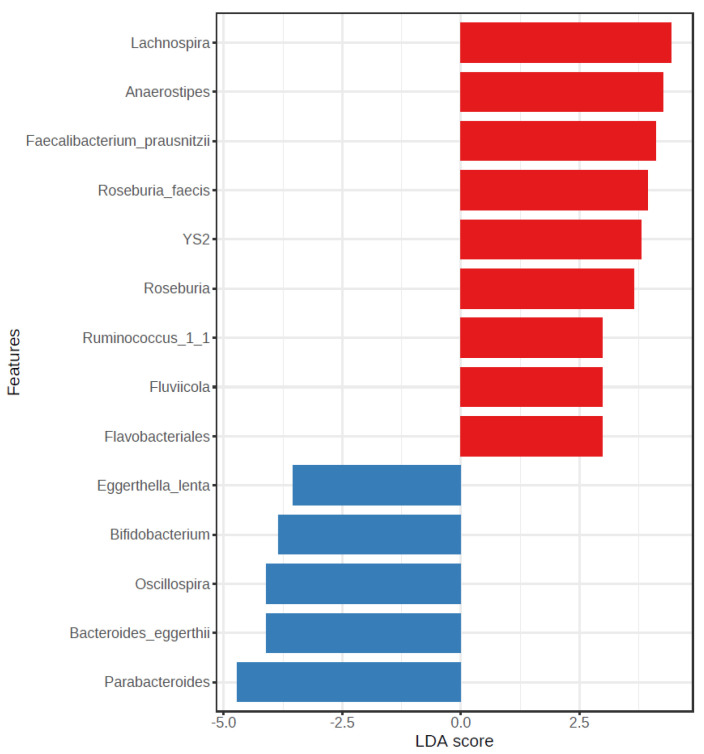
LEfSe analysis at the feature level between the NW group (red) and the OW/OB group (blue) (LDA score > 2). The LDA score (log10) for the most prevalent feature in the OW/OB group is represented on a negative scale, and the LDA score for the most prevalent feature in the NW group is represented on a positive scale.

The LEfSE analysis at the feature level for the determination of potential biomarkers shows significant differences for 14 identified bacteria, which are shown in Figure 5. As it can be observed, bacteria such as *R. faecis* or *F. prausnitzii* appear as biomarkers of NW; this bacterium was previously associated with nutritional adequation to fiber and MUFAs intake, whereas the genus *Oscillospira* was associated with OW/OB individuals who showed a lower consumption of fiber.

## 4. Discussion

### 4.1. Nutritional Analysis

The results obtained from the nutritional analysis showed that the diet followed by the participants of our study is characterized by an elevated consumption of protein and fat, with a high intake of SFA and simple sugars and reduced carbohydrates and fiber. Regarding energy intake, the European Food Safety Authority (EFSA), for its part, recommends an intake of 2000 kcal/day for women and 2500 kcal/day for men [28]. In the present study, the daily energy intake compared with the EFSA suggestions was higher in women, while men adhered correctly to these recommendations. In 2007, a survey on the eating habits of the Galician adult population was conducted with a sample of 3148 individuals [29]. The average intake in the urban sector (results that can be compared with our population sample) for women was 2227 kcal/day. If a comparison is made with the current data obtained in this work, there is only a difference of 20 kcal per day. The opposite happens with men: subjects in this study consumed 75 kcal/day less than the average obtained in the Galician population survey (2577 kcal/day).

Regarding the values for the intake of protein, the average was 16.6 ± 3.5%, which in grams is equivalent to 97.4 ± 15.71 g, a value that exceeds the recommended dietary allowance of 0.8 g/kg body weight/day [30], which for a person weighing about 70 kg would mean an intake of approximately 56 g of protein. On the opposite side of the spectrum to high protein intake is low carbohydrate intake. In 2010, the EFSA’s Technical Commission on Dietetic Products, Nutrition, and Allergies proposed a range of 45–60% as a reference for carbohydrate intake in the European Community [31]. In the case of the Spanish population, the Spanish Society of Community Nutrition (SENC) established nutritional objectives of 50–55% of total energy intake of 50–55% of the total energy [30]. Carbohydrates can be found in five types of food: milk and dairy products, cereals, legumes, fruits, and vegetables [32], and their main function is to provide energy to the body; the human body needs at least 100–150 g/day of this macronutrient to ensure the supply of glucose to glucose-dependent organs and avoid ketosis. From a nutritional point of view, carbohydrates can be divided into two categories. On the one hand, glycemic carbohydrates are considered digested carbohydrates that are absorbed by the small human intestine. On the other hand, dietary fibers are non-digestible carbohydrates that pass into the large intestine [33]. In terms of their influence on the gut microbiome, fiber is considered a key ancestral nutrient that preserves gut ecology, especially by regulating macronutrients and host physiology. Bacterial fermentation of dietary fiber produces key metabolites such as short-chain fatty acids, which are considered beneficial for the host’s health. In addition to its already established properties for glycemic control, findings of gut microbiome correlations with glucose homeostasis can be incorporated into clinical nutrition practice. Targeted dietary fiber interventions on microbiome modulation can offer options to improve glucose control and contribute to personalized nutritional practices [34,35].

In the results presented in this work, the average intake of carbohydrates of our volunteers was de 38.9 ± 7.8% of total energy, which is 11% below the objectives for the Spanish population.

In the case of dietary fiber, the adequacy values are very similar to those for carbohydrates; however, significant statistical differences were observed (*p* = 0.038). It was noted that 84.3% of the volunteers had an intake below the recommendations and only 13.9% were adequate for fiber intake. Dietary fiber has functions such as delaying gastric emptying and maintaining satiety, which may aid in body weight control [35]. It is also associated with a reduction in postprandial peak glucose and insulin, which is a point of interest for people with type 2 diabetes or subjects with glucose intolerance [36]. In terms of BMI, the NW participants in this study had a higher percentage of adequacy (32.5%) than the overweight or obese volunteers (12.0% and 14.3%, respectively). Therefore, this result may indicate the adequacy in fiber intake in participants with NW could be related to a reduction in appetite, which may help the individual to eat less food at subsequent meals, thus balancing daily energy intake [37]. Other studies carried out with the Spanish population, as the ANIBES developed in the year 2019 [38], reported similar results, showing a lower fiber intake of 12.7 ± 5.6 g/day.

Only 2 of the 108 individuals included in the study showed to be fiber intake higher than 50 g/day. High fiber intake can reduce the bioavailability of minerals (iron, calcium, magnesium, and zinc) because phytates, a component present in fiber, can form insoluble compounds with these minerals and affect their absorption at the gastrointestinal level [39]. High fiber consumption is also related to gastrointestinal problems, as fiber can reach the colon intact where it is fermented by the intestinal microbiota of the colon, producing gas that can be accompanied by discomfort with abdominal distension [39]. In terms of the type of bacteria use this fiber, the studies are contradictory. Thus, there are articles reporting an increase in the Bacteroidetes phylum for a diet rich in insoluble fiber, an increase in relative abundance for Proteobacteria for soluble fiber, and for some species of the phylum Firmicutes [40]. Other studies indicate that in humans, it has been reported that Bacteroidetes and Actinobacteria have a positive association with fat but a negative association with fibers, whereas Firmicutes and Proteobacteria show the reverse association [41]. Thus, it seems clear that the metabolism of fiber by the bacteria depends on the type of fiber (soluble and insoluble) and the chemical structure of the fiber (polymerization degree, type of linkages, etc.), as well as the microbiota of the individual. As commented before, the future will allow us to select adequate fiber to develop personalized nutritional practices.

These results, for protein, carbohydrate, and fiber intake, are coincident with previous studies where population diet was analyzed. Thus, a study developed in Buenos Aires (Argentina) [42] evaluated the food intake of 142 adults with an average age of 52 years old, the value for protein intake, in grams, was 97 ± 44 g, 252 ± 117 g of carbohydrates (45% of total energy), and only a 15% of participant achieved the recommendations for fiber. In this study, 49% of the participants showed to be an adequation for refined sugars; however, in the present study, only 13% showed adequacy. In similar studies developed recently in Spain, our results are coincident. Thus, Companys et al. [43] showed, in a study developed with 128 individuals, an elevated protein intake (about 18% of total energy) and a reduced carbohydrate and fiber intake (~38% of energy for carbohydrates and a fiber intake of 25 g/day).

Regarding total lipid intake, 79.6% of the surveyed population exceeded the maximum recommended intake, and only 13.9% accomplished the nutritional objectives of this macronutrient. These data are coincident with the previous studies indicated above [42,43], in which 38.8% and 41% of lipids in terms of total energy, for the two studies, respectively, were reported for the participants.

Fat is one of the macronutrients of greatest interest in the diet, due to its specific nutritional characteristics, as its contribution to the diet is more than twice as many calories per gram (9 kcal/g) as that of carbohydrates or proteins [30]. An adequate intake of total fat provides essential fatty acids and energy to facilitate the absorption of fat-soluble vitamins [44]. A popular belief is that dietary cholesterol is the cause of increased blood cholesterol levels. It is now known that this is not the case, although if consumed in excess, dietary cholesterol can have a detrimental influence on human health [45]. It is worth mentioning that excessive consumption of high-fat foods, accompanied by a sedentary lifestyle, affects body weight and health. In fact, total lipid intake is directly related to BMI and lipid profile; therefore, reducing intake, especially in people with excess body weight, influences weight loss and total and c-LDL cholesterol levels [46].

The SENC recommends a daily intake of three pieces of fruit and two or more pieces of vegetables, adapting to the traditional statement of five portions per day. Of total, 94.4% of the participants surveyed had an intake lower than these recommendations, with an average consumption of 1.8 units. In 2018, through the fruit and vegetable situation report provided by the Spanish Nutrition Foundation, a daily intake of 1.5 pieces of fruit and 1.3 pieces of vegetables was found among the Spanish population [47]. Insufficient fruit and vegetable intake is estimated to be the cause of about 14% of gastrointestinal cancer deaths worldwide, 11% of deaths from ischaemic heart disease, and 90% of deaths from cardiovascular accidents [48]. Scientific research in recent years has focused on the protective role of fruits and vegetables due to their antioxidant potential and their high content of vitamins C, E, and beta-carotene, and other carotenoids, as well as phytochemicals [47].

In general terms, the population studied presented a diet similar to the Western dietary pattern [49]. High intakes of foods rich in fats and sugars reigned supreme, while low intakes of carbohydrates including fiber were conspicuous by their absence. Within these inadequacies, the NW group was those who best met the nutritional targets, compared to the overweight and obese group.

Regarding the data obtained for the nutritional analysis taking into account the BMI, in general, higher adequacy was observed for the NW group. The three groups analyzed presented a similar average intake for nutrients with the exception of fiber (*p* <0.038) and MUFAs (*p* < 0.001). The OW and OB groups showed to consume lower amounts of fiber with outstanding minor adequacy. For MUFAs, the OB group showed lower intake.

Regarding micronutrients. Data obtained in the function of BMI showed that all groups achieved the DRI for vitamin B_12_ and phosphorus. This can be attributed to the high consumption of meat and fish in the diet of the region of Galicia, where the present study was carried out. In addition, phosphorus is found in many ultra-processed foods, which are abundant in a Western diet. Among the micronutrients for which the DRI was not accomplished, vitamin E, folic acid, and calcium showed the lowest adequation. In Spain, the ANIBES report [37] found that 78% of men and 82% of women do not meet adequate vitamin E intake, which coincides with those found in the present work, i.e., 80.5% of men and 95.5% of women did not reach the minimum intake of vitamin E. Folic acid is a micronutrient found in fruits and vegetables, as well as in legumes and other foods such as nuts. The volunteers in the present study reported inadequate intakes of fruit and vegetables, so the low inadequacy could be due to low intakes of these foods.

The high consumption of sodium for the different BMI groups of the surveyed population is noteworthy. The OW and OB showed a 0% of adequation. In Spain, the DRI established by the Spanish Federation of Nutrition, Food and Dietetic Societies (FESNAD) is 1300 mg/day [25]. EFSA considers that an intake of less than 5 g/day (equivalent to 2000 mg Na) represents a healthy salt intake for the general population [50].

In 2010, the average sodium intake worldwide was 3950 mg/day [51]. The results of the ANIBES study in the Spanish population show that the daily intake was 1846 ± 686 mg/day in women and 2219 ± 876 mg/day in men [37]. The results of the present study reveal a sodium intake that is well above the RDI, similar to the global average consumption mentioned above. In both women and men in the Galician population surveyed, the average consumption is three times the RDI (3855.0 ± 1300.3 mg/day and 4156.8 ± 1779.4 mg/day, respectively).

High salt intake may contribute to increased blood pressure, which is one of the main risk factors for cardiovascular disease [52].

### 4.2. Analysis of the Gut Microbiota Composition

Results for alpha- and beta- diversity did not show statistical differences among NW and OW/OB groups, which is coincident with previous studies of dietary intake in lean and obese individuals. In the study carried out with Filipino children [53], no statistical differences were found for alpha- and beta- diversity. The study of Companys et al. [43] with Spanish adults, however, found significant differences in the Chao1 index and beta-diversity.

Regarding the profile of intestinal microbiota for both groups, the studies carried out to date have reported that the gut microbiota in obese individuals is different from that of lean people in terms of the dominant phyla Bacteroidetes and Firmicutes [19,54,55]. In this sense, different studies have indicated that when there is an excess of body fat, the relative abundance of the phyla Firmicutes increased [10]. However, our data showed the contrary since this phylum decreased in the OW/OB group. This can be due to the diet since Firmicutes together with the Bacteroidetes phylum are responsible for complex carbohydrate metabolism in the gut [56]. Since our individuals did not achieve the nutritional recommendations for this macronutrient, especially the OW/OB group, the type of carbohydrates could be determinant for the ratio between this phylum and not rely so much on the body fat. In addition, physical activity can also influence the Firmicutes/Bacteroidetes ratio [57]. Since our OW/OB individuals do not achieve the OMS recommendations of at least 75–150 min of vigorous-intensity aerobic physical activity or an equivalent combination of moderate- and vigorous-intensity activity throughout the week [58], the Firmicutes/Bacteroidetes ratio in this population should also be influenced by this situation.

Although most of the studies indicated that the ratio Firmicutes/Bacteroidetes increased in obesity, other studies are constituent with our data [59,60], and there are also a few studies that did not find any correlation between gut microbiota composition and variations in body weight. In addition, some authors indicate that the evidence does not support a pivotal role for the proportion of Bacteroidetes and Firmicutes, at least at the phylum level, in predisposition to increased body weight, but that diet is responsible to decrease or increase this ratio [61].

Among the bacterial analyzed to obtain differences between the NW and the OW/OB group, it should be pointed out that the results indicated a predominance of *Faecalibacterium*, *Anaerostipes*, and *R. faecis* for the NW group and *Oscillospira* for the OW/OB group.

*Faecalibacterium* is a genus associated with a healthy status, since their species are butyrate producers, taking special relevance to *F. prausnitzii*. Previous studies evaluating intestinal microbiota in OB and NW individuals have reported a decrease in this bacteria in the first one [62], which is one of the most reported lean-associated genera [63]

*Anaerostipes*, a genus belonging to the family *Lachnospiraceae* of the phylum Firmicutes, was also found in significantly higher abundance in NW individuals. Previous studies have shown that this genus was significantly overrepresented in subjects with low inflammatory index [64] and increased in healthy controls when different pathologies have been evaluated as, for example, major depressive disorder [65]. It has also been reported that *Anaerostipes* may protect against colon cancer in humans as a butyric acid producer. In our study, this genus was indicated more abundant in NW individuals.

*R. faecis* is also a species belonging to the family *Lachnospiraceae.* The genus *Roseburia* consists of obligate Gram-positive anaerobic bacteria that are slightly curved, rod-shaped, and motile by means of multiple subterminal flagella. It includes five species: *Roseburia intestinalis*, *R. hominis*, *R. inulinivorans*, *R. faecis*, and *R. cecicola.* Gut *Roseburia* spp. metabolize dietary components that stimulate their proliferation and metabolic activities. They are part of commensal bacteria producing short-chain fatty acids, especially butyrate, affecting colonic motility, immunity maintenance, and anti-inflammatory properties. Modification in *Roseburia* spp. representation may affect various metabolic pathways and is associated with several diseases (including irritable bowel syndrome, obesity, Type 2 diabetes, nervous system conditions, and allergies) [66].

*Oscillospira* was found to increase in OW/OB group. This bacterial genus was associated with constipation in previous studies and predictor of low BMI [67], and it is related in most of the published articles to lean subjects [63,68]; however, our data show the contrary.

For the results obtained from the LefSe analysis, it is remarkable that *Lachnospira*, *Anaerostipes*, F. *prausnitzii*, *R. faecis*, and *Roseburia* together with *YS2* (*Lactobacillus plantarum*), *Ruminococcus*_1_1, *Fluvicola* and *Flavobacteriales* were biomarkers indicative of NW, whereas *Eggerthella lenta*, *Bifidobacterium*, *Oscillospira*, *Bacteroides eggerthii* and *Parabacteroides* were associated with obesity. Some of these bacteria have been discussed above in relation to obesity or lean status. Regarding *L. plantarum*, some studies have reported specific strains that as probiotics can alleviate obesity [69].

*B. eggerthii* was reported increased in obese children in Mexico [70], but it is true that with *Bacteroides* species, the studies indicated it was associated with a healthy status and other ones with dysbiosis or pathologic one.

Regarding *Bifidobacterium*, it has been reported that the *Lactobacillus* and *Bifidobacterium* genera may have a critical role in weight regulation as an anti-obesity effect in experimental models and humans, or as a growth-promoter effect in agriculture depending on the strains [71]. *B. animalis*, for example, has been associated with normal weight status. Although no significant differences have been found between groups, it was noted, in our OW/OB group, that there was a decrease in *B. animalis*, *B. adolescentis*, and *B. longum* and an increment in *B. breve* and *pseudolongum* (data not shown).

Finally, the study presented showed that *P. copri* was more abundant in individuals achieving recommendations for fiber, and this specie was positively correlated with *F. prausnitzii*, *B. ovatus*, *B. caccae*, *B. uniformis*, and *R. faecis*. A negative correlation was found for *B. adolescentis*. *Collinsella aerofaciens*, *Dorea formicigenerans*, *Parabacteroides distasonis*, *Ruminococcus bromii*, and *B. longum*. The bacteria positively correlated with fiber were those more abundant in NW individuals (see Supplementary Material Figure S1).

These results are consistent with other published studies. For example, Lin et al. [72] found that higher fiber intake can affect the composition of the intestinal microbiota, favouring putative beneficial bacteria such as *F. prausnitzii*. Other authors such as Fritsch et al. [73], in a crossover study of 17 participants, found that the abundance of F. prausnitzii increased after 4 weeks on a low-fat, high-fiber diet compared to a standard American diet enhanced with fiber, while Rosés et al. [74], demonstrated a significantly positive correlation between high-fiber intake within the Mediterranean diet context and *R. faecis* abundance. Similarly, studies have shown that after 14 days on a liquid diet supplemented with fiber-rich foods, the abundance of *F. prausnitzii* and *R. intestinalis* is reduced [75].

Therefore, it seems clear that in our study, fiber is the main component determining intestinal microbiota composition. Regarding MUFAs (Figure 4b), P. copri was more abundant in those individuals with adequate consumption, followed by individuals with low consumption, and the lowest abundance was observed for those individuals with high consumption. The species showing a positive correlation with *B. caccae*, *F. prausnitzii*, *R. faecis*, and *B. ovatus*. *B. caccae*, *F.praunsitzii*, and *R. faecis* were significantly reduced in the OW/OB group (see Supplementary Material Figure S1), indicating that these bacteria in addition to the low consumption of fiber could be affected by MUFAs consumption. Previous scientific studies have shown that diets rich in MUFAs do not affect the number of individual bacterial populations but do reduce the number of total bacteria, serum total cholesterol, and LDL-cholesterol values [76]. Since the chemical structure of the fiber is key for the development of one or other bacteria, more studies focusing on the effect of the type of fiber consumed by individuals and focusing on MUFAs should be carried out to clarify this question.

Thus, for our future studies, it should be interesting to evaluate different types of dietary fiber that could be useful to restore this intestinal microbiota imbalance in these OW/OB individuals, test them in a pre-clinical study using an in vitro colonic model, and then select the most appropriate to develop a clinical study with the volunteers. In this clinical study, different biochemistry and genetic parameters, among others, could be investigated to evaluate the effect of this fiber on intestinal microbiota and the host’s health status.

## 5. Conclusions

In this work, a nutritional analysis of individuals from a Northwest region of Spain, Galicia, was conducted to evaluate their adequation to the recommendation. When individuals were compared by BMI, significant differences in fiber and monounsaturated fatty acids (MUFAs) intake were observed, showing higher adequacy for the NW group. Additionally, the analysis of the gut microbiota of the volunteers was also evaluated to obtain statistical differences in 18 ASVs. *Anaerostipes* and *Faecalibacterium* decreased in the OW/OB group, whereas the genus *Oscillospira* increased. A genus was also found in the LEFSe analysis as a biomarker for OW/OB. *R. faecis* was found in a significantly higher proportion of NW individuals and identified as a biomarker for the NW group. Correlation analysis showed that adequation to nutritional recommendation for fiber indicated a higher abundance of *P. copri*, linearly correlated with *F. prautsnitzii*, *Bacteroides caccae*, and *R. faecis*. The same correlation was found for the adequation of MUFA, with. these bacteria being more abundant when the intake was adjusted to or below the recommendations.

## Figures and Tables

**Table 1:** Participants’ diet quality in terms of a caloric lipid profile: adequacy to nutritional objectives for the Spanish population (*n* = 108). Results are expressed as the average ± standard deviation. Diet adequacy is expressed as the number of individuals meeting the nutritional goals and in brackets is expressed as percent.

			DIET ADEQUACY		
CALORIC PROFILE	NUTRITIONAL OBJECTIVES	VALUES OF PARTICIPANTS’ DIET	DÉFICIT	ADEQUATE	EXCESS
Carbohydrates (% Energy)	50–55	38.9 ± 7.8	96 (88.1%)	11 (10.1%)	1 (0.9%)
Fiber (g/1000 kcal)	>14	11.7 ± 7.7	91 (84.3%)	15 (13.9%)	2 (1.9%)
Lipids (% Energy)	30–35	41.8 ± 8.0	7 (6.4%)	15 (13.9%)	86 (79.6%)
**LIPID PROFILE**					
SFA (% Energy)	≤7–8	12.0 ± 3.2	-	13 (0.0%)	95 (88%)
MUFA (% Energy)	20	20.8 ± 16.1	36 (33.3%)	45 (41.7%)	27 (25.0%)
PUFA (% Energy)	5	5.9 ± 3.6	39 (38.0%)	23 (21.2%)	62 (57.4%)
Cholesterol (mg)	<300	347.0 ± 138.6	0 (0.0%)	46 (42.6%)	62 (57.4%)
**OTHERS**					
Water (mL)	2000	2063.3 ± 627.9	49 (45.4%)	59 (54.6%)	0 (0.0%)
Fruits and vegetables	5 portions	1.8 ± 1.5	102 (94.4%)	5 (4.6%)	1 (0.9%)
Sugar (% Energy)	6–10	18.1 ± 7.4	-	13 (12.0%)	95 (88.0%)
Alcohol (g)	≤1 SDU women ≤2 SDU men	10.84 ± 44.43	-	100 (92.6%)	8 (7.4%)

SFA: Saturated Fatty Acids; MFA: Monounsaturated Fatty Acids; PUFA: Polyunsaturated Fatty Acids; SDU: Standard Drink Unit.

**Table 2:** Macronutrient intake for normal weight (NW), overweight (OW), or obese (OB) subjects, and its adequation to the nutritional objectives for the Spanish population (*n* = 108). Caloric profile, lipidic profile, and others are shown. Results are expressed with mean ± standard deviation. Diet adequacy is expressed as the number of individuals meeting the nutritional goals and in brackets is expressed as percent.

		NW (*n* = 40)		OW (*n* = 35)		OB (*n* = 33)		
CALORIC PROFILE	Nutritional Objectives	Mean ± SD	Intake Adequacy: *n* (%)	Mean ± SD	Intake Adequacy: *n* (%)	Mean ± SD	Intake Adequacy: *n* (%)	*p* Value
Carbohydrates (% Energy)	50–55%	40.3 ± 7.1	5 (12.5%)	37.4 ± 7.7	1 (3.0%)	38.8 ± 8.6	4(11.4%)	0.274
Lipids (% Energy)	30–35%	40.6 ± 6.3	6 (15.0%)	43.6 ± 9.6	5 (15.2%)	41.9 ± 7.5	5 (14.3%)	0.280
Fiber (g/1000 Kcal)	>14	14.2 ± 9.5	13 (32.5%)	12.5 ± 10.14	4 (12.0%)	9.1 ± 2.9	5 (14.3%)	0.038
**LIPID PROFILE**								
SFA (% Energy)	≤7–8	11.8 ± 2.8	3 (7.5%)	11.9 ± 3.0	4 (12.1%)	15.5 ± 8.1	0 (0.0%)	0.559
MUFA (% Energy)	20	20.3 ± 5.7	10 (25.0%)	19.4 ± 5.7	12 (36.4%)	6.5 ± 5.3	5 (14.3%)	<0.001
PUFA (% Energy)	5	5.8 ± 2.1	12 (30.0%)	6.5 ± 5.3	19 (57.6%)	5.4 ± 2.7	6 (17.4%)	0.874
Cholesterol (mg)	<300 mg	340.3 ± 137.7	19 (47.5%)	348.0 ± 129.8	11 (33.0%)	354.1 ± 152.1	10 (28.6%)	0.915
**OTHERS**								
**Water (mL)**	2000 mL	2305.36 ± 487.6	23 (57.5%)	2128.0 ± 682.2	20 (57.4%)	2098.1 ± 736.4	16 (48.5%)	0.751
**Fruits and vegetables**	5 portions	1.9 ± 1.5	3 (7.5%)	1.3 ± 1.4	1 (2.8%)	2.2 ± 1.5	1 (3.0%)	0.785
**Sugar**	6–10	20.0 ± 9.0	1 (2.5%)	19.0 ± 7.1	5 (14.3%)	16.0 ± 5.1	7 (21.2%)	0.632
Alcohol (g)	≤1 SDU women ≤2 SDU men	3.8 ± 9.8	39 (97.5%)	4.0 ± 8.7	32 (97.0%)	3.9 ± 8.8	31 (88.6%)	0.298

SFA: Saturated Fatty Acids; MFA: Monounsaturated Fatty Acids; PUFA: Polyunsaturated Fatty Acids; SDU: Standard Drink Unit.

**Table 3:** Micronutrient intake for normal weight (NW), overweight (OW), or obese (OB) subjects, and its adequation to the Dietary Reference Intakes (DRI) for the Spanish population (*n* = 108). Diet adequacy is expressed as the number of individuals meeting the DRI and in brackets is expressed as percent.

	NW (*n* = 40)		OW (*n* = 35)		OB (*n* = 33)		
Vitamins	Average ± SD	Intake Adequacy *n* (%)	Average ± SD	Intake Adequacy *n* (%)	Average ± SD	Intake Adequacy *n* (%)	*p* Value
Thiamine (mg)	1.5 ± 0.8	32 (80.0%)	1.9 ± 0.9	31 (88.6%)	1.4 ± 0.3	29 (87.9%)	0.009
Riboflavin (mg)	1.7 ± 0.5	30 (75.0%)	2.1 ± 0.6	31 (88.6%)	1.7 ± 0.4	27 (81.9%)	0.007
Niacin (mg)	30.5 ± 10.7	30 (75.0%)	40.4 ±14.5	16 (45.7%)	31.6 ± 7.7	25 (75.8%)	0.0005
Vitamin B_6_ (µg)	2.0 ± 0.8	35 (87.5%)	2.3 ± 1.1	33 (94.3%)	2.0 ± 0.6	30 (90.9%)	0.159
Folic acid (µg)	281.3 ± 126.3	13 (32.5%)	278.3 ± 105.2	10 (28.6%)	255.3 ± 74.6	8 (24.2%)	0.537
Vitamin B_12_ (µg)	6.1 ± 6.0	40 (100%)	6.4 ± 2.5	35 (100%)	5.6 ± 2.8	33 (100%)	0.697
Vitamin C (mg)	151.2 ± 87.7	35 (87.5%)	166.2 ± 79.6	33 (94.3%)	160.7 ± 68.5	33 (100%)	0.738
Vitamin A (µg)	798.4 ± 603.1	24 (60.0%)	750.5 ± 452.7	19 (54.3%)	745.0 ± 406.8	17 (51.5%)	0.868
Vitamin D (µg)	2.9 ± 3.2	8 (20.0%)	4.6 ± 6.0	11 (31.4%)	4.5 ± 6.5	11 (33.3%)	0.321
Vitamin E (mg)	8.3 ± 8.1	5 (12.5%)	7.8 ± 9.9	4 (11.4%)	5.9 ± 5.6	2 (6.1%)	0.416
**Minerals**							
Calcium (mg)	719.8 ± 297.1	7 (17.5%)	841.2 ± 415.0	12 (34.3%)	792.9 ± 348.4	10 (30.3%)	0.331
Magnesium (mg)	325.7 ± 134.9	23 (57.5%)	368.9 ± 95.8	23 (65.7%)	308.8 ± 81.0	15 (45.5%)	0.063
Potassium (mg)	3086.7 ± 904.1	22 (55.0%)	4044.5 ± 1135.9	26 (74.3%)	3511.8 ± 745.6	24 (72.7%)	0.0001
Phosporus (mg)	1415.5 ± 500.5	38 (95.0%)	1716.9 ± 472.9	34 (97.1%)	1436.8 ± 380.6	32 (97.0%)	0.009
Iron (mg)	16.7 ± 9.0	23 (57.5%)	18.3 ± 5.8	29 (82.9%)	14.0 ± 2.8	21 (63.6%)	0.035
Iodine (µg)	184.4 ± 150.3	16 (40.0%)	347.5 ± 197.4	25 (71.4%)	292.5 ± 153.6	25 (75.8%)	0.0002
Zinc (mg)	24.4 ± 78.7	29 (72.5%)	14.1 ± 3.5	34 (97.1%)	10.8 ± 2.9	27 (81.8%)	0.0445
Sodium (mg)	2610.9 ± 677.5	6 (15.0%)	5609.5 ± 1219.7	0 (0.0%)	3877.1 ± 346.0	0 (0.0%)	<0.0001

## Data Availability

The data presented in this study are available on request from the corresponding author.

## Supplementary Materials

The following supporting information can be downloaded at: https://www.mdpi.com/article/10.3390/nu15153418/s1, Figure S1: Correlation analysis. Figures show the obtained results for the correlation analysis between identified bacterial species and BMI of individual (NW in red and OW/OB in green, groups) Red lines showed a positive correlation among bacterial species whereas blue lines showed a negative correlation; Table S1: Participants’ diet quality by sex, in terms of caloric profile and lipid quality: adequacy to nutritional objectives for the Spanish population.; Table S2: Micronutrient intake and adequation to the Dietary Reference Intakes (DRI) for the Spanish population in women and men.
