# Supplementary material for: Compliance with Nutritional Recommendations and Gut Microbiota Profile in Galician Overweight/Obese and Normal-Weight Individuals
Source: Nutrients. 2023 Aug 1;15(15):3418. doi: 10.3390/nu15153418 (PMC10420825; doi:10.3390/nu15153418)
Supplement: Supplementary file 1 [file nutrients-15-03418-s001.zip › nutrients-2508167-supplementary.pdf]

Supplementary Material

Table S1. Participants' diet quality by sex, in terms of caloric profile and lipid quality: adequacy to nutritional objectives for the Spanish population.

		**Females (n=67)**		**Males (n=41)**		
**CALORIC PROFILE**	**Nutritional objectives**	**Average ± SD**	**Intake adequacy: n (%)**	**Average ± SD**	**Intake adequacy: n (%)**	***p* Value**
Protein (% Energy)	10 – 15 %	16.6 ± 3.5	27 (40.3%)	16.7 ± 3.5	14 (34.1%)	0.317
Carbohydrates (% Energy)	50 -55 %	38.9 ± 7.8	7 (10.4%)	38.7 ± 3.8	3 (7.3%)	0.197
Lipids (% Energy)	30 – 35 %	41.8 ± 8.0	10 (14.9%)	42.1 ± 7.9	5 (12.2%)	0.360
Fiber (g/1000 kcal)	>14	11.7 ± 7.7	10 (14.9%)	11.5 ± 7.7	5 (12.2%)	0.137
**LIPID PROFILE**						
SFA (% Energy)	≤7 – 8	11.32 ± 2.4	9 (13.43%)	13.06 ± 3.9	4 (9.8%)	0.005
MUFA (% Energy)	20	20.8 ± 16.1	24 (35.8%)	20.9 ± 16.3	19 (46.3%)	0.635
PUFA (% Energy)	5	6.3 ± 4.3	12 (17.6%)	5.2 ± 1.5	12 (29.3%)	0.121
Cholesterol (mg)	< 300 mg	347.0 ± 138.6	28 (41.8%)	347.0 ± 138.6	13 (31.7%)	0.074
**OTHER**						
Water (ml)	2000 ml	2075.2 ± 642.9	36 (53.7%)	2119.3 ± 612.4	23 (56.1%)	0.752
Fruits and vegetables	5 portions	1.7 ± 1.4	3 (4.5%)	1.8 ± 1.7	2 (4.8%)	0.844
Sugar (% Energy)	6 - 10	17.9 ± 5.9	6 (8.9%)	18.3 ± 9.5	7 (17.1%)	0.791
Alcohol (g)	≤ 1 SDU women<br>≤ 2 SDU men	3.3 ± 9.8	62 (92.5%)	4.6 ± 6.6	38 (92.7%)	0.559

Results expressed with mean ± standard deviation; SFA: Saturated Fatty Acids; MFA: Monounsaturated Fatty Acids; PUFA: Polyunsaturated Fatty Acids. Diet adequacy is expressed as the number of individuals meeting the nutritional goals. Expressed as n (%).

Table S2. Micronutrient intake and adequation to the Dietary Reference Intakes (DRI) for the Spanish population in women and men

	**Females (n=67)**				**Males (n=41)**				
	**RDI**	**UL**	**Average ± SD**	**Intake adequacy n (%)**	**RDI**	**UL**	**Average ± SD**	**Intake adequacy n (%)**	***p Value***
**Thiamine**	1	-	1.4 ± 0.4	58 (86.6%)	1.2	-	1.87 ± 1.0	34 (89.9%)	0.003
**Riboflavin (mg)**	1.3	-	1.7 ± 0.4	54 (80.6%)	1.6	-	2.03 ± 0.6	34 (82,9%)	0.001
**Niacin (mg)**	14	35	31.6 ± 10.2	48 (71.6%)	17	35	37.98 ± 13.9	23 (56,1%)	0.007
**Vitamin B6 (µg)**	1.2	25	1.9 ± 0.6	61 (91.0%)	1.5	25	2.41 ± 1.1	38 (92,7%)	0.003
**Folic acid (µg)**	300	1000	262.0 ± 87.5	20 (29.8%)	300	1000	289.4 ± 129.1	11 (26.8%)	0.192
**Vitamin B12 (µg)**	2	-	5.7 ± 4.6	67 (100%)	2	-	6.62 ± 3.3	41 (100%)	0.228
**Vitamin C (mg)**	60	-	158.5 ± 79.0	62 (92.5%)	60	-	159.7 ± 80.4	39 (95.1%)	0.940
**Vitamin A (µg)**	600	3000	711.3 ± 432.9	38 (56.7%)	700	3000	856.8 ± 583.3	22 (53.7%)	0.141
**Vitamin D (µg)**	5	100	4.0 ± 5.4	21 (31.3%)	5	100	3.9 ± 5.3	9 (22.0%)	0.950
**Vitamin E (mg)**	15	300	5.9 ± 3.5	3 (4.5%)	15	300	10.0 ± 12.0	8 (19.5%)	0.009
**Calcium (mg)**	1000	2500	756.7 ± 321.2	13 (19.4%)	900	2500	822.0 ± 404.6	16 (39.0%)	0.356
**Magnesium (mg)**	300	-	318.3 ± 88.9	37 (55.2%)	350	-	361.2 ± 135.0	19 (46.3%)	0.772
**Potassium (mg)**	3100	-	3423.8 ± 849.6	43 (64.2%)	3100	-	3695.5 ± 1236.5	29 (70.7%)	0.179
**Phosporus (mg)**	700	-	1436.7 ± 3810	65 (97.0%)	700	-	1655 ± 575.2	39 (95.1%)	0.019
**Iron (mg)**	15	-	15.1 ± 4.8	32 (47.8%)	9	-	18.6 ± 8.8	41 (100%)	0.008
**Iodine (µg)**	150	600	259.5 ± 158.9	43 (64.2%)	150	600	288.0 ± 211.8	23 (56.1%)	0.427
**Zinc (mg)**	7	25	10.9 ± 3.3	62 (92.5%)	9.5	25	26.6 ± 77.3	28 (68.3%)	0.098
**Sodium (mg)**	1300	-	3855.0 ± 1300.3	4 (6.0%)	1300	-	4156.8 ± 1779.4	2 (4.9%)	0.312

RDI: Recommended Daily Intake; **UL:** Tolerable Upper Intake Level; **-:** No adequate data to derive a UL. Results expressed as average ± standard deviation (SD). Differences in continuous variable were examined using T-student for parametric variable and using Wilcoxon rank-sum for non-parametric variables. Statistical significance was set at p value < 0.05.

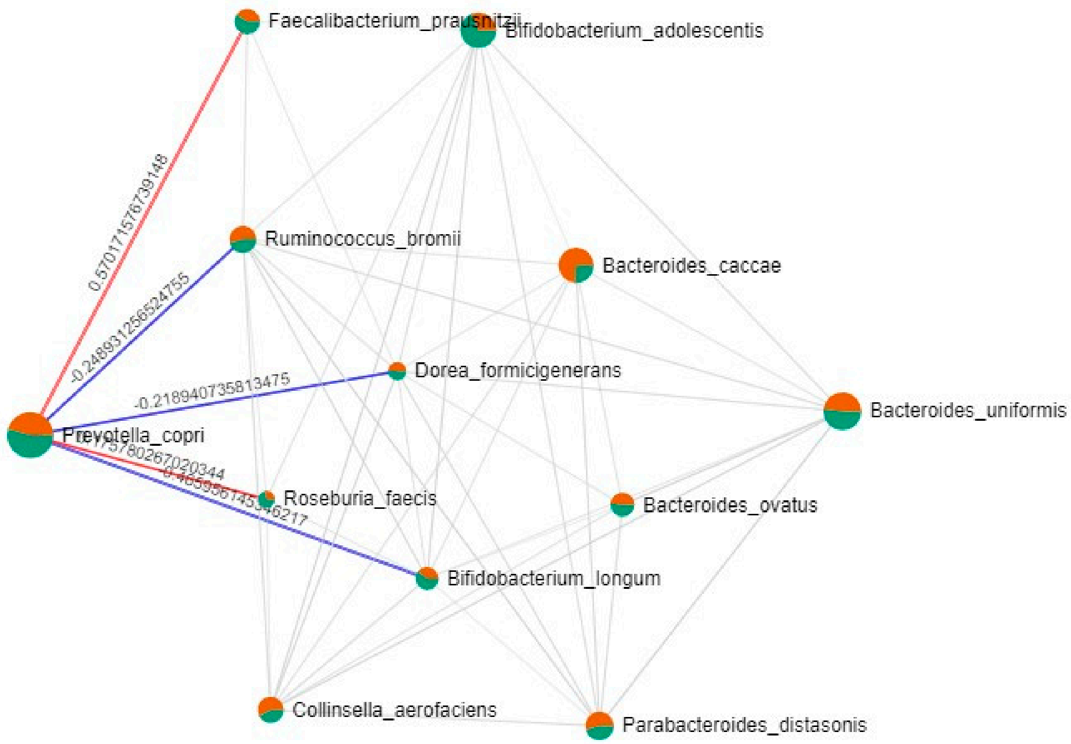

Figure S1. Correlation analysis. Figures show the obtained results for the correlation analysis between identified bacterial species and BMI of individual (NW in red and OW/OB in green, groups) Red lines showed a positive correlation among bacterial species whereas blue lines showed a negative correlation.
